# Translation process of the tested Rome IV diagnostic questionnaire for functional gastrointestinal disorders into Saudi-Arabian Arabic: A mixed-methods approach

**DOI:** 10.12669/pjms.40.8.9126

**Published:** 2024-09

**Authors:** Elham A. Aljaaly, Mai A. Khatib

**Affiliations:** 1 Elham A. Aljaaly, Department of Clinical Nutrition, Faculty of Applied Medical Sciences, King Abdulaziz University, Jeddah, Saudi Arabia. Medical Nutrition Therapy Unit, King Abdulaziz University Hospital, King Abdulaziz University, Jeddah, Saudi Arabia; 2 Mai A. Khatib, Department of Clinical Nutrition, Faculty of Applied Medical Sciences, King Abdulaziz University, Jeddah, Saudi Arabia. Food, Nutrition, and Lifestyle Unit, King Fahd Medical Research Centre, King Abdulaziz University, Jeddah, Saudi Arabia. Obesity and Lifestyle Unit, King Abdulaziz University Hospital, King Abdulaziz University, Jeddah, Saudi Arabia

**Keywords:** Children, diagnostic questionnaire, functional gastrointestinal disorders, ROME IV, translatability, Arabic, Saudi Arabia

## Abstract

**Objective:**

A report on the cross-cultural adaptation and validation process of the Rome IV Diagnostic Questionnaire for children aged four years and over into Saudi-Arabian Arabic for use in assessing the prevalence of functional gastrointestinal disorders in children in Saudi Arabia.

**Method:**

A mixed-methods approach was used in translating the 60-item original English version of the questionnaire. The process included four steps followed by a cognitive debriefing and was guided by the Rome Foundation. The questionnaire was tested for practicability with 10 participants of children aged four years and older. The whole study took place between October 2020 and April 2021.

**Results:**

The original questionnaire repeated information on areas of pain experienced by children, which did not show up in the backward, English, translation. The back-translated version occasionally provided medical expressions that were then explained between parentheses in plain English, for example, dyspepsia (burning feeling). The expert panel indicated that all questionnaire items reached the set 90% agreement level, confirming that the questionnaire is fully understandable and valid for use. Preliminary testing with 10 participants (four years and older) revealed functional constipation to have the highest prevalence among the participants (40%, n=4), followed by irritable bowel syndrome (20%) and abdominal migraine (20%).

**Conclusion:**

This study provides a detailed report on the translation process of the tested ROME- IV Diagnostic Questionnaire for children aged four years and over into Saudi Arabic following Rome Foundation guidelines. The results of the preliminary test should encourage researchers and clinicians in Saudi Arabia to utilize the tool for non-invasive diagnosis of functional gastrointestinal disorders in children.

**Abbreviations:**
DQ: Diagnostic questionnaire,
FGIDs: Functional gastrointestinal disorders,
RF: Rome Foundation,
TT: Translation team.

## INTRODUCTION

Functional gastrointestinal disorders (FGIDs) are presently identified as disorders of gut-brain interaction. They are a cluster of disorders categorised by gastrointestinal symptoms including motility disturbances, visceral hypersensitivity, and alterations in the mucosa, immune function, and gut microbiota.^1^,^2^ Suggestions for the classification of FGIDs were considered in 1990^3^, after which the Rome Foundation (RF) started issuing criteria to facilitate the diagnosis and identification of populations with FGIDs.^4^ Evidence has supported practitioners’ reliance on non-invasive diagnostic approaches in evaluating dysfunction in children.^5^-^8^ The Rome IV Diagnostic Criteria are non-costly, non-invasive, symptom-based diagnostic criteria that help to identify children with FGIDs. Diagnosis involves going through a checklist of subjective symptoms, including onset, duration, and frequency, with either the child or the parents.^9^ Examples of paediatric FGIDs defined by the Rome IV diagnostic criteria are functional abdominal pain disorders, which include functional dyspepsia, abdominal migraine, irritable bowel syndrome, and functional abdominal pain, if not specified.^10^ The Rome criteria are continually updated, and updates include the introduction of new names, modifications to previously considered diagnoses, and the definition of new FGIDs.^11^,^12^ In 2016, the Rome IV replaced its predecessor, the Rome III.^13^

Methods used in the tool-translation process in cross-cultural research are diverse and vary in quality. Reviews have confirmed the need to document the reliability, precision, and validity of these methods.^14^,^15^ The Rome IV Diagnostic Questionnaire (DQ) for the examination of the prevalence of FGIDs in children was originally developed in English, and is completed mainly by children’s parents.^16^,^17^ However, if researchers need to use the questionnaire in a different language and with a different culture, the ROME criteria require them to translate and validate the DQ in the new language.^18^ Studies on several cultural adaptations of the criteria have shown that the translate to other language versions produced adequate diagnostic precision, supporting their use for the diagnosis of FGIDs in children.^17^,^19^-^21^

Cross-cultural prevalence studies usually follow observational designs and select samples that are convenient and representative of the target country.^14^ Testing of the Saudi Arabic version of the non-invasive tool facilitated the diagnosis of several FGIDs in children, including functional dyspepsia, constipation, irritable bowel syndrome, and aerophagia.^22^

Considering the current dearth of information and comprehensive studies on diagnostic approach and prevalence of FGIDs among Saudi children, and the lack of a Saudi Arabic version of the internationally accepted Rome IV DQ, the researchers in the present study recognised the crucial role of having a Saudi Arabic version. The tool will support researchers in epidemiological research exploring the prevalence of FGIDs in school-aged children. The authors also intended to provide clinicians with a tool that can be completed by either them or the children’s parents to aid in diagnosis and in improving quality of life and health status of children with FGIDs. This paper describes the cross-cultural adaptation process of the Rome IV DQ for FGIDs in children aged four years and older into Saudi Arabic along with the pilot study of the translated questionnaire carried out with 10 participants of children aged four years and older, particularly after confirming its passable diagnostic precision and support in diagnosing FGIDs in Saudi children.^22^

## METHODS

The study was conducted from October 2020 to April 2021, during which online invitations were utilized to accommodate COVID-19 restrictions, facilitating participant access. The translation process occurred earlier in conjunction with obtaining ethical clearance.

**Fig. 1:**
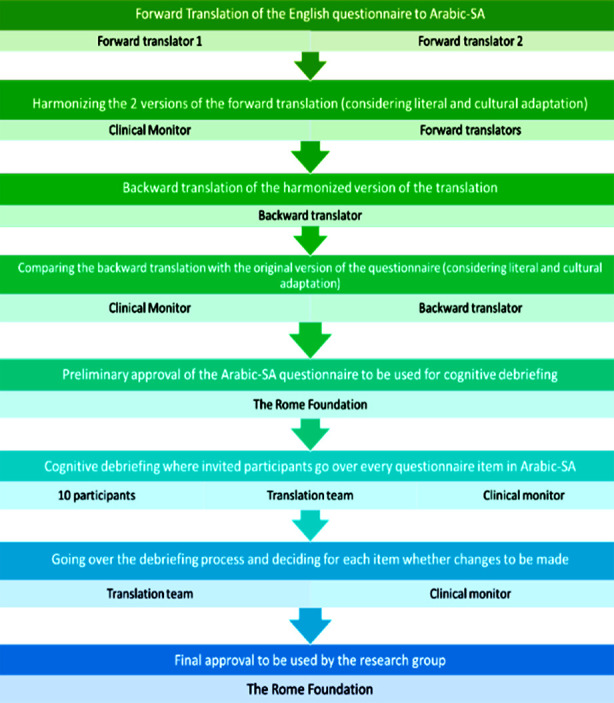
The process of translating the ROME IV Diagnostic Questionnaire into Saudi Arabic.

The researchers conducted an online search of the literature and contacted the Rome Foundation, the original developer of the ROME IV DQ for FGIDs in children, and confirmed that no Saudi Arabic version was available. Next, a *translation team* (TT) was assembled and permission was obtained, with signed agreement, to develop an Arabic translation following the RF’s rigorous pre-established guidelines, the steps of which are outlined in Fig.1.

### The translation process:

### The Original Rome IV Questionnaire:

The Rome IV DQ for children is a self-reported tool that is filled out by parents to subjectively measure specific gastrointestinal symptoms in children. The tool includes 60 items tackling different areas where gastrointestinal symptoms can be felt (such as the oesophagus, stomach, small intestine, and colon) and assessing frequency. These symptoms may or may not apply to all children. The DQ consists of five sections: above the belly button, around or below the belly button, bowel movements, nausea and vomiting, and other gaseous symptoms such as burping and air swallowing. The RF provided the TT with the questionnaire’s scoring algorithm to facilitate the identification of functional disorders.

### The Translation Process:

This was a mixed-methods approach in which quantitative and qualitative data were collected in multiple steps:

- forward translation by two translators
- comparison of the two forward-translated versions;
- backward translation of the harmonised translated version;
- comparing the backward translation with the original questionnaire. The last steps included a cognitive debriefing process to validate the Saudi Arabic version and the final approval of the DQ (Fig.1).

The TT consisted of two clinical nutrition health professionals with experience in gastrointestinal disorders who can speak and write in both English and Arabic perfectly. Both team members were familiar with the questionnaire’s use as a measure in different events, particularly for paediatric gastrointestinal disorders.

The RF directed the TT throughout the translation process in the selection of translators for each stage, based on its guidelines and the chosen translators’ working knowledge. The RF also appointed a local clinical monitor to accompany and monitor the whole process. The selected clinical monitor was an expert physician in gastroenterology fluent in the target language and originally from Saudi Arabia.

### The forward translation:

This step aimed to produce a cultural, rather than literal, translation of the DQ in the target language, and was achieved by two independent bilingual professional translators who were native in the target language and residing in Saudi Arabia. The TT explained the concept of the tool to both translators to make them more familiar with it and help them translate it more accurately.

### Harmonising the two versions of the forward translation:

The two resulting Arabic versions of the questionnaire were harmonised into a single version by considering literal translations and cultural adaptation. This process was carried out by the forward translators and reviewed by the clinical monitor.

### Backward translation of the harmonised version:

The resulting Arabic version of the DQ was back-translated into English by an independent professional medical translator who is a native English speaker. The backward translator was not involved in the first step of translation in any way. The selected translator was also unaware of the meanings of the concepts that the questionnaire measures. The purpose of this step was to ensure accuracy of the forward translation by revealing any misunderstood or uncertain wording that may have resulted.

### Comparing the backward translation with the original questionnaire:

The clinical monitor and the backward translator compared the DQ’s back translation with the original version, item-by-item, on two dimensions: language similarity (literal translation) and comparability of interpretation (cultural adaptation). Following this step, the TT obtained final approval from the RF to use the Saudi Arabic version of the questionnaire and move on to the final step, the cognitive debriefing process.

### Cognitive debriefing process to validate the Saudi Arabic version of the Rome IV Diagnostic Questionnaire:

The cognitive debriefing process was carried out, from 26 February to 12 March 2021, to make cross-cultural comparisons and verify the validity of the translation process. For this step, the RF suggested that the TT assembles an expert panel of five parents to give their feedback on language and cultural issues. Invitations were sent to health care professionals with clinical nutrition background who were also mothers of children aged four years and older and who spoke both Arabic and English. The TT asked each member independently to explain every question back to the TT and suggest changes in wording where applicable and useful.

During this step, the final Saudi Arabic version of the Rome IV DQ was reviewed by the expert panel in terms of clarity, cultural adaptation, language level, and acceptability. This step required familiarity with the cognitive debriefing process, which involves the TT going over every DQ item with the expert panel members to get their feedback on comprehensibility and whether changes were needed.

### Decision on the validated tool:

The translators and the clinical monitor went over the debriefing process to decide which questionnaire items required changes, based on expert panel members’ feedback. Items were approved if they received 90% agreement, or over, from reviewers. Questions that received an agreement level below 90% were modified by the TT until they could achieve the 90% minimum. The final version of the DQ was then sent to the RF for final approval.

### Testing the tool for practicability:

The TT took an additional step of testing the DQ with 10 participants of children aged four years and older to assess its practicability after being approved for use. This step was carried out from 25 March to 20 April 2021.

### Participants

Invitations were sent to 10 non–health professionals who are parents of children aged four years or older to fill out the Arabic version of the questionnaire. Participants who met the criteria for recruitment and agreed to participate were selected.

The Rome IV tool was shared electronically through Zoom meetings and WhatsApp to grant easy access to participant responses. Data—which included 60-items (quantitative) and one open-ended question for suggestions (qualitative)—were collected using online interactive interviews, where the researchers were virtually present to respond to any raised question or comment. The idea of digitizing the questionnaire came about due to the limitations that were in place during the COVID-19 pandemic (quarantine and physical contact restrictions). This step further enabled the TT to evaluate the practicability of the translated DQ in assessing the prevalence of FGIDs in Saudi children using the data collected from the 10 participants aging four years and older.

### Ethical approval and consent to participate:

This study commenced following review of the research protocol by the research team and the receipt of ethical approval from the Research Ethics Committee at the Faculty of Applied Medical Science of King Abdulaziz University (Reference no.: FAMS-EC2021-03), obtained on February 16, 2021. Consent was obtained from all participants in the preliminary test and voluntary participation was explained to all participants before they joined the study.

### Statistical analysis:

Data are presented in numbers and percentages. Microsoft Excel (Version 2019) and GraphPad Prism 9 (GraphPad Software, San Diego, CA, USA) were used to analyse data and produce graphs. Scoring of individual questions followed RF guidelines to identify disorders and their prevalence.

## RESULTS

### Translation process of the Rome IV Diagnostic Questionnaire:

The key in-country person and the forward translators provided by the project manager had background information about the conceptual basis of the measure. Following the project manager’s instructions, they produced colloquial translations intended for the general public. The tool only targeted children, even though the requested response was from the parents. Therefore, it was necessary to keep wording compatible with certain reading levels or ages.

Words and concepts that had an unclear equivalent in the original language were modified in the back translation. Comparing the back translation with the original version of the questionnaire revealed some identical words used to explain pain or discomfort but also a few minor linguistic and cultural concerns such as necessary clarifications for medical terms (e.g., dyspepsia = feeling of burning). The original version repeated some information on pain areas, which was not seen in the backward translation.

The forward-backward translation process was used to detect differences and similarities between the English source language and the target Saudi Arabic versions. This was achieved through comparison performed by an independent bilingual translator who was a native English speaker, who served as a monolingual judge in the target language of the Saudi Arabian community. Finally, an item-by-item comparison was carried out comparing literal translations with cultural adaptations.

Overall, the described process revealed that the resulting Arabic version of the ROME IV DQ is an adequate translation that uses very similar language and expressions (making it easy to use) and that it should deliver the desired results. Following this comparison, the RF agreed to using the produced Saudi Arabic version for the cognitive debriefing step.

### Cognitive debriefing:

This step addressed the expert panel’s thoughts on each questionnaire item (N=5 responses) and the need for changes in order to finalize the process and obtain final approval of the translation from the RF. Each member on the expert panel independently checked all 60 items of the questionnaire in both languages and explained what they thought of each questionnaire item. Three comments were obtained from the interactive virtual interviews (N=5). The comments concerned the nature of the Rome IV DQ and its length. One respondent (20%) thought that the time needed to complete the questionnaire was too long. Another respondent (20%) commented on the targeted age group of children, specifically children 4- to 5-years old, saying that some of them may still be wearing nappies and going through toilet training and may soil their clothes with faeces unrelated to FGIDs.

Therefore, some of the questions on bowel habits may not correspond to the actual case scenario. The third comment (20%) was a recommendation to interview participants face-to-face in order to prevent confusion with some common symptoms. However, there were no comments or feedback on the translation of the questions (i.e., whether any questions needed clarification or re-translation), which was the purpose of carrying out this step.

Based on this, the DQ was declared fully understandable, and no changes were required since none of the comments reached the 90% level of agreement. This approach allowed the TT to ensure that the translated items retained the original meaning as well as the absence of confusion.

These results were reviewed and discussed by the TT and the clinical monitor and then forwarded to the RF, where the final product of the questionnaire in Saudi Arabic was approved.

### Preliminary testing of the Arabic version:

**Fig.2:**
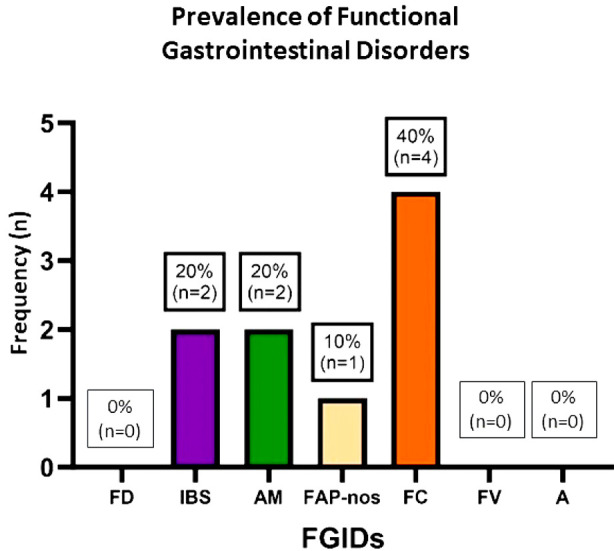
Prevalence of functional gastrointestinal disorders among children of the 10 participants (aged four years and older) who filled out the Arabic version of the diagnostic tool. Fig.2 shows 9/10 cases were positive for FIGDs.

Among the 10 collected responses, 40% (n=4) reported being for boys and 60% (n=6) for girls. All of the children were Saudi nationals. The highest prevalence of FGIDs was 40% (n=4) for functional constipation, followed by 20% (n=2) each for irritable bowel syndrome and abdominal migraine, and 10% (n=1) for functional abdominal pain-not otherwise specified. Table-I, Fig.2 show the prevalence of FGIDs among the tested 10 children, of which 9/10 cases were positive for FIGDs.

**Table-I:** Prevalence of FGIDs among the children of parents (N=10) who participated in the preliminary test.

Functional gastrointestinal disorder	No. (%)
Functional dyspepsia	0 (0%)
Irritable bowel syndrome	2 (20%)
Abdominal migraine	2 (20%)
Functional abdominal pain–nos	1 (10%)
Functional constipation	4 (40%)
Functional vomiting	0 (0%)
Aerophagia	0 (0%)

Nos, not otherwise specified.

## DISCUSSION

In this paper, the TT discusses the steps and results of the translation process for the Rome IV DQ concerning guidelines of the RF and other related evidence. The TT also discusses results of the adapted tool’s preliminary testing with 10 participants of children aged four years and older. The main results of the translation process were presented in the Nutrition 2021 Live Online Conference of the American Society of Nutrition.^23^

Translation processes can face difficulties that can portend their authority^24^, making it a challenge for any TT to produce a culturally adapted tool that retains the meaning and intent of the original while also being culturally relevant and understandable. The researchers managed to include adequate information to establish and validate the quality of the translation process into Saudi Arabic in order to, consequently, enable them to gain official approval to translate the tool. The aim of translating the Rome IV DQ into Saudi Arabic was to provide a validated diagnostic tool in the Arabic language for non-invasive diagnosis and the conduct of prevalence studies in children with FGIDs.

The translation process included various steps starting with the forward translation and ending with the cognitive debriefing, in which the source and target versions of the tool were studied for linguistic features, including literal versus cultural meaning. The researchers followed the pre-established rigorous methodological process and strict guidelines of the RF that were sent to the TT before beginning the translation.^21^,^25^ The forward translation of the English version into Saudi Arabic was performed by two professional translators who are native Arabic speakers and residents of Saudi Arabia, as recommended by the RF guidelines and other studies, to better reflect the tone in the target language.^25^,^26^

After the forward translation, an independent translator fluent in the original language performed a backward translation to ensure accuracy of the forward translation and avoid process bias. Previous reports have shown significant benefits to performing back translation as opposed to forward-only translation, because the back translation allows for comparison between the source and the target languages.^27^ Furthermore, there is a risk that the back-translation would not be aligned with the source language, as previous research has shown, which can lead to errors in the translation or errors being ignored and remaining in the translated version.^28^ During the current translation process, the review of the back translation allowed for the introduction of alterations to specific words and concepts throughout the tool when necessary. This was exceptionally useful for instances where either of the two versions included ideas or words that are socially or culturally unacceptable or that could be difficult to communicate in other languages.

To deal with situations where no similar concept was available, which could result in misinterpretation, the RF appointed a clinician monitor in the relevant medical field who is a native speaker of Saudi Arabic to critically review the work, at two stages of the process, in order to consider literal and cultural adaptation:

- harmonising the two versions of the forward translation;
- comparing the backward translation with the original version. This process is consistent with reported guidelines for the translation of data collection tools.^28^

When translating material into different languages, inconsistency between translators could result. This, however, can be avoided and solved between the selected translators; otherwise, an unbiased bilingual translator, who was not involved in the previous translations, can be asked to carry out the job.^14^ Thus, the backward translation of the DQ was compared with the original English version, a process that can easily evaluate the equivalence of meaning between the source and target texts.

The RF requires translators translating their DQ into other languages to conduct a cognitive debriefing process as part of their standardized guidelines. This step is of intense importance in the paediatric age group, because children could use diverse words at different ages for the same construct, and this is also affected by gender.^29^ Translators can use this process to ensure that translated items retain the same meaning as the original and to avoid any misunderstanding in the translated questionnaire.^30^ Based on this, the TT of this project asked the five parents (members of the expert panel) to individually explain each question back to them and suggest changes in wording if they found it valuable.

The TT carefully selected participants for the cognitive debriefing using convenience sampling from Saudi health professionals in the field of clinical nutrition who have a solid background and knowledge of the Saudi community. They were invited to participate in the debriefing process, and since they were in the same field as the TT, they were easily approached. The interpretability of the data collected in this stage depends largely on accurate responses from participants. The developer of the tool requested that qualitative data be collected from respondents (the five members of the expert panel) through face-to-face interviews.

However, because of the COVID-19 pandemic and restrictions in place regarding physical contact, an electronic version of the tool was created to provide easy access to the expert panel’s responses. At the same time, in order to make the interviews interactive, the TT interviewed the respondents using virtual programs such as Zoom meetings and social media channels such as WhatsApp. Therefore, the TT was actively present with the respondents to obtain more information on what they thought about each item in the questionnaire, what their corresponding responses meant, and to answer any questions that may arise. There were no comments from the 5 participants in the preliminary test regarding the translated items, indicating that the translated questionnaire items correspond to those in the original and are consistent with the respondents’ interpretation of those items.^31^ This stage was followed by final approval from the RF for use of the Saudi Arabic translation of the DQ in future local research and as a diagnostic tool by clinicians.

To take the decision on the validated tool**,** the TT and the clinical monitor went over the debriefing process and decided whether to make changes to the questionnaire items based on the participants’ feedback, in terms of the comprehensibility. This tool was developed as a screening tool for diseases that may not clinically show dysmorphology, as an inclusion criterion in clinical trials, and to support epidemiological surveys.^32^

Following recommendations from the literature^33^, the TT conducted a preliminary testing of the DQ to test the translated tool for practicability with 10 participants of children aged four years and older, which allowed for further testing to assess prevalence of FGIDs in a lager sample of children (N=59).^22^ The same approach (virtual interviews) used for the validation process was used for data collection in the preliminary test. The results of the preliminary testing of the tool showed that among.

Among the measured FGIDs, functional constipation was the highest prevalent (40%, n= 4 out of 10), supporting reports that it is common in Saudi Arabia.^34^ This does not, however, agree with the results reported by Khatib and Aljaaly in 2022 who used the same translated tool with 59 participants. Their results showed that functional dyspepsia was the most prevalent FDIG in children (11.8%), followed by functional constipation (5%), then irritable bowel syndrome (1.6%) and aerophagia (1.6%).^22^ Interestingly, both functional dyspepsia and aerophagia were not prevalent among participants of the current study.

Collectively, FGID prevalence results from both studies using the Saudi Arabic translation of the tool can benefit future research. The new Saudi Arabic version can help practitioners in this area in defining various FGIDs among children in Saudi Arabia. It could also be used to raise parents’ awareness about FGIDs in children, which can go undiagnosed whilst they need special treatment, further supporting the importance of translating such tools for local use.

To the best of the researchers’ knowledge, this work is the first to have been done in Saudi Arabia. The authors conducted this work in support of good practice in translating and adapting non-invasive diagnostic tools for children with FGIDs.

### Limitations:

The cognitive debriefing process and the preliminary testing of the DQ were carried out during the COVID-19 pandemic, as result of which the TT had to rely on virtual meetings and digitize the DQ.

The selection of Saudi Arabian Arabic for translation may not be clear to readers, who may question why this specific dialect was chosen and how it relates to the cultural context of the study. Therefore, the authors clarification about this decision, as differed to a more generalized Arabic dialect, was informed by the authors’ country of research and the targeted study sample. The translation process was guided by the Rome Foundation, which considers linguistic and cultural factors to ensure the questionnaire resounds with the intended audience. However, it is acknowledged that cultural distinctions may vary across Arabic-speaking regions, potentially influencing respondents’ interpretations. While efforts were made to ensure unaffected and relevant translation, the potential impact of cultural differences on respondent comprehension cannot be guided.

The study did not provide a copy of the translated survey as a supplementary file or within the document. The inability to include the translated survey because the translation process and distribution of the survey are fully controlled by the RF, the main developer of the original version. As per copyright regulations, the RF retains the rights to both the original and translated tools, and any copies must be obtained directly from their website. Consequently, this limits the survey tool accessibility by the readers.

The authors referred to select out-of-date references in reporting this work that were, nonetheless, crucial and extremely relevant to the focus of the study. These include reports on the diagnosis of FGIDs and RF reports on the tool’s development and translation.

## CONCLUSION

To be able to compare responses between populations of different languages and/or cultures, researchers need to ensure that the questionnaires used in different languages use the same constructs and identical measure. The translation process of the 60-item ROME IV DQ followed RF guidelines and was performed and monitored by an experienced translation team of native speakers in the target language (Arabic) and residents of the target country (Saudi Arabia). The availability of bilingual professionals throughout the translation process, including the TT, the translators, the clinical monitor, and the respondents who participated in the cognitive debriefing facilitated the conduct of the crucial processes at all stages.

This study provides a template for the translation and reporting of an existing non-invasive diagnostic tool into Saudi Arabic, bearing in mind that the Rome IV DQ for children (four years and older) was initially developed in English-speaking countries and that the translated version is needed for researchers who intend to collect data from respondents residing in Saudi Arabia. Although the translation process was time consuming and relatively costly, it was a valuable step required to ensure that the translated version of the questionnaire is equivalent to the original version. To prepare the tool for use in future local epidemiological research, the tool was tested with 10 participants with who aged four years and older, and the results confirmed the tool’s practicability for exploring the prevalence of FDIGs in Saudi children.

### Abbreviations:

**FGIDs**, functional gastrointestinal disorders, **FD**, functional diarrhoea, **IBS**, irritable bowel syndrome, **AM**, abdominal migraine, **FAP**–nos, functional abdominal pain-not otherwise specified, **FC**, functional constipation, **FV**, functional vomiting, A, Aerophagia.

### Author Contributions:

**EA:** Took the lead in writing the manuscript.

**MK:** Analysis and reporting the data. Both authors provided critical feedback and helped shape the study, analysis, and the manuscript. Both authors reviewed and approved the final draft of the questionnaire.

Both authors carried out the translation process and prepared the manuscript.
